# Transgenerational Exposure to Environmental Tobacco Smoke

**DOI:** 10.3390/ijerph110707261

**Published:** 2014-07-16

**Authors:** Xavier Joya, Cristina Manzano, Airam-Tenesor Álvarez, Maria Mercadal, Francesc Torres, Judith Salat-Batlle, Oscar Garcia-Algar

**Affiliations:** 1 Unitat de Recerca Infància i Entorn (URIE), Institut Hospital del Mar d’Investigacions Mèdiques (IMIM), Barcelona 08003, Spain; E-Mails: jjoya@imim.es (X.J.); jsalat@imim.es (J.S.-B.); 2 Paediatric Unit, Hospital del Mar, Barcelona 08003, Spain; E-Mails: 61059@parcdesalutmar.cat (C.M.); 60809@parcdesalutmar.cat (A.-T.A.); 60810@parcdesalutmar.cat (M.M.); 61041@parcdesalutmar.cat (F.T.)

**Keywords:** environmental tobacco smoke, non-conventional matrices, nicotine, cord blood, free-smoke policies, newborn, children

## Abstract

Traditionally, nicotine from second hand smoke (SHS), active or passive, has been considered the most prevalent substance of abuse used during pregnancy in industrialized countries. Exposure to environmental tobacco smoke (ETS) is associated with a variety of health effects, including lung cancer and cardiovascular diseases. Tobacco is also a major burden to people who do not smoke. As developing individuals, newborns and children are particularly vulnerable to the negative effects of SHS. In particular, prenatal ETS has adverse consequences during the entire childhood causing an increased risk of abortion, low birth weight, prematurity and/or nicotine withdrawal syndrome. Over the last years, a decreasing trend in smoking habits during pregnancy has occurred, along with the implementation of laws requiring smoke free public and working places. The decrease in the incidence of prenatal tobacco exposure has usually been assessed using maternal questionnaires. In order to diminish bias in self-reporting, objective biomarkers have been developed to evaluate this exposure. The measurement of nicotine and its main metabolite, cotinine, in non-conventional matrices such as cord blood, breast milk, hair or meconium can be used as a non-invasive measurement of prenatal SMS in newborns. The aim of this review is to highlight the prevalence of ETS (prenatal and postnatal) using biomarkers in non-conventional matrices before and after the implementation of smoke free policies and health effects related to this exposure during foetal and/or postnatal life.

## 1. Introduction

Second-hand tobacco smoke (SHS) consists of exhaled smoke as well as side-stream smoke that is released from the burning cigarette between inhalations and it has a very similar composition [1]. SHS is referred to as “environmental” tobacco smoke (ETS) [2]. The composition of SHS changes as it becomes diluted with ambient air, with its distribution in the environment and as it interacts over time with compounds found in the environment. However, no matter how SHS changes in the environment, it still contains significant levels of nicotine (NIC). NIC concentrations in the air in homes of smokers range on average from 2–10 μg/m^3^ [2].

Tobacco is also a major burden on people who do not smoke. As developing individuals, children are particularly vulnerable to the negative effects of SHS, which may even occur before birth [3,4,5]. Furthermore, they are unable to influence their own degree of exposure. ETS, side-stream, or SHS are associated with significant health risks such as cancer, heart disease, asthma, and/or respiratory illnesses. In the field of perinatal epidemiology, children’s exposure to tobacco constituents during foetal development through the placenta, when the environment is inside the uterus, and via exposure during childhood is perhaps the most ubiquitous and hazardous of children’s environmental exposures [6].

## 2. Materials and Methods

The following eligibility criteria were used: (a) clinical studies; (b) laboratory-based investigations; (c) studies published only in English language letters to the Editor, historic reviews, commentaries and case-reports were excluded.

PubMed/MedLine (National Library of Medicine, Washington, DC, USA) databases were searched from 1988 up to and including June 2014 using different combinations of the following key words: “environmental tobacco smoke”, “passive”, “nicotine”, “secondhand” and “smoking”. Titles and abstracts of studies identified using the above-described protocol were screened by the authors and checked for agreement. Full-texts of studies judged by title and abstract to be relevant were read and independently evaluated by the authors with reference to the inclusion and exclusion criteria. Reference lists of articles retrieved from the initial search were hand-searched to identify any studies that could have remained unidentified in the previous step.

## 3. ETS and Health Effects in Neonatal and Paediatric Population: An Overview

Prenatal SHS exposure puts unborn babies at risk for (1) stillbirth and preterm delivery [7]; (2) growth retardation [7,8]; (3) congenital anomalies [8,9]; and (4) respiratory infections and asthma in childhood [4]. Worldwide, at least 40% of children are regularly exposed to SHS after birth, additionally predisposing them to upper and lower respiratory infections as well as asthma [10].

A meta-analysis on passive smoking during pregnancy and foetal health estimated that exposure of non-smoking pregnant women to SHS reduces mean birth weight by 33 g or more, and increases the risk of a birth weight below 2500 g [5,11]. Similar to this study, many others reported a significant association between SHS and premature birth [12,13,14,15,16].

In addition, there is documented and unequivocal epidemiological evidence to support an association between active smoking during pregnancy and low birth weight (LBW) and growth retardation [17]. On the whole, data from several studies show that there is a dose-response effect of smoking on both mean birth weight and the incidence of LBW infants with an adjusted OR of 2.3 (95% CI 2.0–2.04) for smoking pregnant women compared to non-smokers [18,19]. A recent meta-analysis of 16 studies reported an OR for LBW of 1.22 (95% CI 1.10–1.35) [20].

Newborns of women who smoke have increased risks of congenital anomalies such as orofacial clefts [21]. A recent meta-analysis of 12 studies reported an association between ETS and an increased risk of having an infant with a congenital anomaly (OR 1.17; 95% CI 1.03–1.34) [8].

Active maternal smoking during pregnancy has been associated with a higher risk of behavioural disorders in children; a few cohort studies have studied its specific effects on the cognitive abilities of pre-schoolers by measuring smoking data prospectively. The disorders range from personality temperament to neuropsychiatric outcomes such as attention disorders (e.g., attention deficit hyperactivity disorder (ADHD)) [22]. Maternal smoking habits were associated with lowered cognitive development of children at age 4 years [23]. ETS is also associated with an increased risk of psychiatric morbidity. A recent meta-analysis found that NIC studies indicated a greater risk of ADHD-related disorders among children whose mothers smoked during pregnancy more than 10 cigarettes/day (OR, 1.85 (95% CI 1.74–1.96); N = 175.869) [24].

Finally, the most studied health effect of ETS during prenatal and postnatal life has been the altered respiratory function. It is well known that prenatal maternal smoking and postnatal ETS lead to a dose-dependent increase in respiratory morbidity in infants and children. Exposure to ETS causes asthma, wheezing, cough, bronchitis, pneumonia, and impaired pulmonary function [25]. Several studies have shown a reduced lung function in newborns exposed passively to tobacco smoke during pregnancy [26,27,28,29]. Similarly, many studies demonstrated that parental smoking status has an important impact on asthma and wheezing illnesses in infants and children. A study conducted in 3-year-old children who were exposed both prenatally and postnatally to ETS reported increased prevalence of wheezing (OR, 1.14) when compared with children born to non-smoking parents [30]. Also, the incidence of lower respiratory illness (LRI) increases with parental smoking [31,32,33,34]. Long-term exposure to ETS creates a state of permanent inflammation and an imbalance in the lipid profile that leads to lipid accumulation in the blood vessels of the heart and aorta [35]. Children with long-term exposure to ETS may have an elevated risk for the development of premature coronary artery disease [36]. Hypertension is the leading risk factor for cardiovascular disease. Newborn infants of smoking parents show symptoms of cardiovascular stress hyperactivity. Simonetti *et al.* [37] found that both systolic (+1.0 mmHg) and diastolic (+0.5 mmHg) blood pressure were higher in children of smoking parents. In healthy preschool children, parental smoking is an independent risk factor for higher blood pressure, adding to other familial and environmental risk factors.

## 4. Biological Matrices to Evaluate ETS Prevalence in Paediatric Population

Prenatal tobacco exposure has been usually assessed using a self-reported maternal questionnaire [38,39,40]. However, difficulties in recognizing smoking behaviour or recalling smoking exposure, or changes in smoking habits during gestation could bias these assessments. In Mediterranean countries, with a high prevalence of young female smokers [41,42], questionnaires could be even less valid [43,44]. In addition, pregnant women, conscious of the risks of tobacco smoke for the foetus, may be reluctant to admit active (or passive) tobacco smoke exposure due to social pressure, guilt, or embarrassment [42,45].

Similar to other drugs of abuse, licit or illicit, biological markers specific to tobacco smoke have been identified in the past two decades to prevent reporting bias. NIC and its major metabolite, cotinine (COT), have been used as biomarkers for SHS in conventional (blood and urine) [46,47] and non-conventional matrices (saliva, meconium, and hair) [48,49,50] (Table 1).

**Table 1:** Biological matrices for the evaluation of prenatal and postnatal exposure to environmental tobacco smoke.

Biological Matrix	Detection Window	Collection	Biomarker Levels for the Different Exposure Groups
**Prenatal evaluation**			
**Maternal hair**	Months	Easy and non-invasive. Hair washing to remove external contamination	Hair NIC is more precise that urine COT. NIC (ng/mg) of non-exposed: 0.53; highly exposed and smokers: 5.95 [48,51]
**Cord serum**	Hours to days	Easy and non-invasive (at birth)	COT levels in cord serum indicate fetal exposure to tobacco. COT (ng/mL) of no-exposure: <LOD; low-exposure: 1–14; medium-high exposure: >14 [52]
**Neonatal urine**	1 to 3 days before delivery	Easy and non-invasive	Close correlation between NIC and COT maternal and neonatal concentrations. COT (ng/mL) of non-exposed: 1.9 [53]; and exposed: 170.5 [54]
**Amniotic fluid**	Months (1st and 2nd trimesters)	Invasive collection procedure	Human fetus exposed to higher NIC concentrations than the smoking mother [55]
**Neonatal meconium**	2nd and 3rd trimesters of pregnancy	Easy and non-invasive. May be delayed until 3 days	Mean COT (ng/g) levels from: non-exposed to ETS: 6.0 [56] and highly exposed: 42.6 [56]
**Postnatal evaluation**			
**Oral Fluid**	0.5–36 h	Easy and non-invasive. Performed under direct observation	Salivary COT is more sensitive than NIC. COT (ng/mL) of non-exposed: 0.44; exposed: 3.38 [57]
**Hair**	Months	Easy and non-invasive. Hair washing to remove external contamination	Hair NIC is more precise that urine COT. NIC (ng/mg) of non-exposed: 0.53; highly exposed: 5.95 [51]
**Teeth**	Years	Easy and non-invasive. Requires pulverization and organic washing.	NIC indicates cumulative exposure to tobacco smoke. Mean NIC (ng/g) of non-smokers parents: 15.0 [58]; both parents smokers: 42.3 [59]

NIC is the primary addictive component of tobacco and it is the major constituent of cigarettes. NIC presents a half-life of approximately 2–3 h in blood, followed by urinary excretion [60]. About 80% of NIC is transformed to COT [61]. COT presents a longer biological half-life in comparison to NIC and it has been found to be directly related to daily cigarette consumption [62]. The measurement of COT in blood, saliva, or urine, has been used to support epidemiologic findings of causal relationships with SHS exposure [63].

### 4.1. Biological Matrices for the Evaluation of Prenatal Exposure to Environmental Tobacco Smoke

The concentrations in foetal blood can be indicative of transplacental passage of NIC and its metabolites during pregnancy. Concentrations of NIC metabolites in cord blood are in the order of ng/mL (the concentrations are two or three orders of magnitude lower than those detected in the amniotic fluid) [55]. Different cut-offs (14 and 21.5 ng/mL) have been proposed to differentiate between passive and active smokers. To differentiate between exposure and non-exposure to ETS, a cut-off value of 1 ng/mL has been proposed [40,52,64]. Garcia-Algar *et al.* [42,45] investigated the association between COT in cord serum and in maternal and newborn urine samples. Cord serum COT appeared to be the most sensitive biomarker for foetal exposure to smoking at the end of pregnancy, distinguishing not only active smoking from passive smoking, but also exposure to ETS from non-exposure.

Traditionally, urine has been considered the specimen of choice for neonatal drug testing for several reasons: even though urine collection is difficult, it is superior to the invasive serum collection and COT analysis in neonatal urine is an easy, rapid, and low-cost test [53]. Köhler *et al.* determined NIC, COT and trans-3′-hydroxicotinine in maternal and neonatal urine. A close correlation (which did not depend significantly on the time of urine collection) was found between maternal and neonatal NIC and COT concentrations [54]. A disadvantage of urine is that the time window of detection is short, reflecting drug use only a few days before delivery. Analysis of the neonatal urine may produce false-negative results depending not only on the time of the last ingestion of the drug by the mother but also on the length of time after birth when the specimen was collected [42]. COT but not NIC concentrations in mothers’ plasma, breast milk, and infants’ urine reflected the smoking habits during pregnancy [43]. However, in other studies this relationship was not corroborated, after studying 429 mothers and their newborns, measuring COT concentrations in neonatal urine and cord serum [42]. Recently, meconium became the specimen of choice for detecting drug exposure in neonates. Meconium can be collected between 1 and 5 days after birth, and its collection is easy and non-invasive. Drug concentrations in meconium are generally higher than in urine because of accumulation over several months of gestation. Recently, Gray TR *et al.* [56] demonstrated that there was a significant correlation between NIC and metabolites in meconium and a decrease in head circumference.

### 4.2. Biological Matrices for the Evaluation of Postnatal Exposure to Environmental Tobacco Smoke

Oral fluid is an interesting ETS marker for acute consumption that occurred in the hours previous to the collection, and is less invasive and more cost-effective than blood. Transfer of tobacco smoke constituents and their metabolites from blood to saliva occurs primarily by passive diffusion. Jarvis *et al.* [57] developed different methods for the quantification of COT in saliva. Using a cohort of 569 non-smoking school-children, they measured COT concentration in saliva. The study showed that when neither parent smoked, the mean concentration was lower than that for either parent or the sum of the concentration for both the parents. Hair testing has been considered the “gold standard” to assess chronic ETS. Because COT accumulates in hair during hair growth, it is a unique measure of long-term, cumulative exposure to tobacco smoke. The major potential advantage of the hair test, when compared with saliva and serum measurements, is its ability to reflect long-term exposure (months) rather than short-term exposure (hours or days). It is estimated that hair grows at approximately 1 cm/month [65]. Therefore, hair can be analyzed in monthly, 1-cm segments, creating a “calendar” of NIC exposure [51]. Pichini *et al.* [66] measured NIC and its principal metabolite in 24 children aged 3–36 months attending a nursery school in the suburbs of Rome. For the first time, in the paediatric population, NIC measurement in hair was used to categorize different statuses of chronic exposure to ETS. Al-Delaimy *et al.* [67,68] measured NIC hair levels in 117 children and evaluated the effect of avoidance strategies such as smoking outside the household. Levels of NIC in hair of children reportedly exposed to smokers were higher than levels of unexposed children. A disadvantage of hair analysis is the need to use analytical methods based on chromatography techniques coupled with MS methods that require qualified personnel. Teeth demonstrated the potential of this biological matrix as an important deposit of exogenous substances, which can accumulate both in the pulp and in the calcified tissues. Garcia-Algar *et al.* [58] analyzed NIC and COT in deciduous teeth from children of both non-smoking and smoking parents. The results support NIC analysis in teeth as a promising non-invasive tool for monitoring and categorizing cumulative exposure to ETS from fetal life [59].

## 5. Policies to Diminish the ETS during Pregnancy and in the Entire Childhood

Smoke-free policies have been expanded worldwide since the WHO encouraged countries to follow Article 8 of the Framework Convention on Tobacco Control (FCTC) [2] to protect people from SHS. Legislation has been widely implemented in indoor public places, workplaces, and public transportation [69]. Several countries have implemented legislations requiring all enclosed workplace and public places to be free of SHS [70]. Ireland was the first country with comprehensive smoke-free legislation implemented in 2004. Since then, countries like Norway, New Zealand, Italy, Spain, Uruguay, England and many provinces or states in Canada, the USA or Australia [64,71] followed. This law includes health recommendations against smoking and recommendations for regulation of tobacco smoking in public places with wide exemptions in bars, restaurants and night clubs.

Data from different investigations carried out worldwide on the effects of smoking bans on ETS demonstrate clearly that SHS has decreased after the implementation of smoke-free legislation although in some cases (hospitality venues) high SHS levels have been found [72]. Interventions to reduce exposure to SHS have been found to reduce the incidence of cardiovascular diseases [73,74,75,76] but only few studies have examined the effect on pregnancy outcomes [15,77,78].

The scientific results showed a decreasing trend of exposure to SHS and an increase in the number of individuals not-exposed to SHS [79], including prenatal non-exposure to SHS [64,76,78,80]. Results from a cohort study performed in our hospital located in Barcelona city (Spain) examined the effect of Spanish legislation in three populations (1998–2006) of newborns recruited at different times during the implementation of the law [78]. These results demonstrate a decrease in SHS evidenced by a higher percentage of samples with cord blood COT < 1 ng/mL. The mean cord blood COT levels in the newborns from all the study groups were: 3.21 ng/mL in 1996–1998, 0.80 ng/mL in 2002–2004 and 0.44 ng/mL in 2008. The percentage of no prenatal SHS exposure (cord blood COT 0.2–1 ng/mL) showed an increase compared to the previous groups (1996–1998 and 2002–2004) while the percentages of both: low (1.1–14 ng/mL) and very high (>100 ng/mL) prenatal SHS exposure showed a decrease in the number of babies recruited after the implementation of the smoke-free law. The change could be explained as a combination of several factors: negative messages in communication media and the public awareness derived from them; the implementation in 2005 of Spanish smoke-free legislation; and the increase in the tobacco price for the fiscal modification. Another fact that can explain this decrease could be the implementation of programs to help smoking pregnant women to quit (program “EmbaràsSense Fum”) in Catalonia. These programs included specific training and free NIC replacement treatment for pregnant smoking women throughout pregnancy [81].

In other countries, such as Italy, the joint action of legislation and prevention through campaigns against smoking has turned Italy into one of the countries with a lower percentage of non-smoking pregnant women exposed to ETS and the lowest percentage of women smoking during pregnancy, as shown in an urban area. In Italy, Franchini M *et al.*, using questionnaires and cord blood COT, demonstrated that 13.5% of newborns were exposed to ETS and 7.7% were exposed to active maternal smoke [64]. In England, Sims M *et al.* using questionnaires and saliva COT, obtained similar results [76,82].

Despite that, few scientific articles have analyzed the effect of this decrease in the SHS in the paediatric population. McKay DF *et al.* [83] determined the impact of the Scottish’s legislation on preterm delivery. His data shows a reduction in the risk of preterm delivery (−11.72%, 95% CI −15.87, −7.35, *p*<0.001) and a reduction in the prevalence of current smoking which fell from 25.4% before legislation to 18.8% after legislation but this evaluation was not performed using the appropriate markers to assess SHS exposure (e.g., airborne NIC and COT in saliva). These results have now been confirmed in several follow-up studies [77,84,85].

## 6. Conclusions

Tobacco smoke, active or passive, is the most prevalent substance used during pregnancy worldwide. It is well established that active maternal smoking (and ETS) during pregnancy impairs foetal growth and shortens gestation producing deleterious birth outcomes. Since 2005, different countries, including Spain, have approved different smoke-free laws that ban smoking in all public places and workplaces including restaurants and pubs in order to protect people from high levels of exposure. Moreover, the development of new techniques to determine NIC and its metabolites in non-conventional matrices such as saliva, cord blood or hair has been a tremendous step in assessing the occult prevalence of ETS in newborns and children. Using objective biomarkers, different studies have demonstrated a decrease in the level of exposure after the implementation of smoke-free laws. Due to the decrease in the prevalence of SHS exposure, there are a few publications which highlighted the normalization of birth weight and preterm birth incidence but more studies are needed to confirm these preliminary observations.
